# Crowdsourcing for translational research: analysis of biomarker expression using cancer microarrays

**DOI:** 10.1038/bjc.2016.404

**Published:** 2016-12-13

**Authors:** Jonathan Lawson, Rupesh J Robinson-Vyas, Janette P McQuillan, Andy Paterson, Sarah Christie, Matthew Kidza-Griffiths, Leigh-Anne McDuffus, Karwan A Moutasim, Emily C Shaw, Anne E Kiltie, William J Howat, Andrew M Hanby, Gareth J Thomas, Peter Smittenaar

**Affiliations:** 1 Cancer Research UK, 407 St John Street, London EC1V 4AD, UK; 2 Cancer Research UK Cambridge Institute, University of Cambridge, Robinson Way, Cambridge CB2 0RE, UK; 3 Southampton CRUK Centre, University of Southampton Faculty of Medicine, Tremona Road, Southampton SO16 6YD, UK; 4 CRUK/MRC Oxford Institute for Radiation Oncology, University of Oxford, Roosevelt Drive, Oxford OX3 7DQ, UK; 5 Cancer Research UK Leeds Centre, University of Leeds, Beckett Street, Leeds LS9 7TF, UK

**Keywords:** cancer, immunohistochemistry, tissue microarray, crowdsourcing, biomarker, pathology

## Abstract

**Background:**

Academic pathology suffers from an acute and growing lack of workforce resource. This especially impacts on translational elements of clinical trials, which can require detailed analysis of thousands of tissue samples. We tested whether crowdsourcing – enlisting help from the public – is a sufficiently accurate method to score such samples.

**Methods:**

We developed a novel online interface to train and test lay participants on cancer detection and immunohistochemistry scoring in tissue microarrays. Lay participants initially performed cancer detection on lung cancer images stained for CD8, and we measured how extending a basic tutorial by annotated example images and feedback-based training affected cancer detection accuracy. We then applied this tutorial to additional cancer types and immunohistochemistry markers – bladder/ki67, lung/EGFR, and oesophageal/CD8 – to establish accuracy compared with experts. Using this optimised tutorial, we then tested lay participants' accuracy on immunohistochemistry scoring of lung/EGFR and bladder/p53 samples.

**Results:**

We observed that for cancer detection, annotated example images and feedback-based training both improved accuracy compared with a basic tutorial only. Using this optimised tutorial, we demonstrate highly accurate (>0.90 area under curve) detection of cancer in samples stained with nuclear, cytoplasmic and membrane cell markers. We also observed high Spearman correlations between lay participants and experts for immunohistochemistry scoring (0.91 (0.78, 0.96) and 0.97 (0.91, 0.99) for lung/EGFR and bladder/p53 samples, respectively).

**Conclusions:**

These results establish crowdsourcing as a promising method to screen large data sets for biomarkers in cancer pathology research across a range of cancers and immunohistochemical stains.

Personalised medicine is reliant on the determination of markers and genetic profiles that facilitate targeting of therapies to those who will benefit the most. Achieving this aim depends on translational studies from clinical trials whereby success of the new agent, modality or regime is correlated with profiles observed in the target tissues. By their nature these studies generate large tissue sets. Progress therefore depends on pathologists having sufficient time for research, which is becoming increasingly difficult in an environment of increasing workload and severe financial constraints on healthcare and research across the world. In this context, the future of medical research is critically dependent upon innovation to improve productivity and increase efficiency (UK Accelerated Access Review). We hypothesised that contributions from the general public – also known as ‘crowdsourcing' – can have a role in accelerating biomedical research. Here we explore its application in the field of immunohistochemistry (IHC) scoring in human cancer tissue samples.

Histopathologists have a key role in both medical diagnostics and translational research. While demand for histopathologists has never been higher, in most part due to increases in cancer cases (+30% in the UK since the late 1970s; Cancer Research UK, ‘Cancer incidence for all cancers combined', 2013), there has been a precipitous decline in the academic histopathology workforce. In the US, the proportion of pathologists in the population is predicted to drop by 35% between 2010 and 2030 (Robboy *et al*, 2013), whereas the UK has seen a 60% drop in academic pathologists between 2000 and 2012 (Wilkins, 2015). Many of the solutions proposed to address this deficit can only be realised in the long-term, whereas more resource is required immediately to ensure an ongoing contribution of tissue sample interrogation to translational research. Machine learning promises to automate many routine evaluations (Bolton *et al*, 2010; Wilbur, 2014; Bouzin *et al*, 2015; Howat *et al*, 2015), but commonly requires large, validated data sets for its development. Crowdsourcing can provide such data sets in addition to solving an immediate need for analytical resource.

Crowdsourcing (or citizen science) is the provision of services by distributed members of the general public. Such services take many forms, including problem solving, nature surveys, environmental monitoring, and data processing (Ranard *et al*, 2014). Crowdsourcing has existed for close to two centuries but experienced a surge in popularity over the past decade, particularly facilitated by internet and mobile technologies. Current scientific applications include the classification of images of distant galaxies (Lintott *et al*, 2008), puzzle games designed to create a three-dimensional visual representation of the brain (Seung and Burnes, 2012), discovering tertiary structures of proteins (Cooper *et al*, 2010), as well as bug hunting and genome sequence analysis (Kawrykow *et al*, 2012; Good and Su, 2013; Rallapalli *et al*, 2015).

Here we crowdsourced the analysis of tumour samples prepared as tissue microarrays (TMAs). Tissue microarrays facilitate high-throughput molecular analysis of tissue samples to investigate associations between tumour-specific protein expression and clinical outcomes (Giltnane and Rimm, 2004). Although automated analysis of TMAs has proven to be effective for specific screening protocols, particularly in breast cancer (Turbin *et al*, 2008; Bolton *et al*, 2010; Konsti *et al*, 2011; Howat *et al*, 2015), it was also observed that algorithms underperform on less well-established markers such as cytokeratin (CK) 5/6 and epidermal growth factor receptor 1 (EGFR/HER1; Howat *et al*, 2015). In the same study, 20–25% of samples had to be manually excluded from the analysis. This suggests a synergy between crowdsourcing and automated analysis, whereby manual exclusion and scoring could precede the training of an automated algorithm. A key feature in this approach is that crowdsourcing can compensate for slight deficits in accuracy through the sheer volume of data it can process.

We previously developed Cell Slider (www.cellslider.net) to invite members of the public to score breast cancer TMA cores for oestrogen receptor (ER) staining (Candido Dos Reis *et al*, 2015). We observed that users tended to overestimate the number of cancer cells in an image, compromising the accuracy of IHC scores. This lack of specificity in Cell Slider was most likely due to a minimal level of instruction provided prior to scoring the samples, as well as a restrictive interface showing only a small portion of a TMA sample, preventing users access to an overview of the tissue. Here we present a novel crowdsourcing interface developed to improve upon Cell Slider. First we set out to test the effects of feedback-based training and provision of annotated example images on the ability of scorers to detect cancer in a sample. We then used this improved tutorial to assess performance in cancer detection in four sample types selected as being of interest to academic pathologists. Finally, we examined the accuracy of IHC scoring in a lung cancer sample with membrane expression of EGFR, and bladder cancer with nuclear expression of p53.

## Materials and methods

### Participant recruitment and ethics

Participants were recruited through e-mails to individuals registered for non-pathology Cancer Research UK crowdsourcing projects. Newsletters and advertising were used to recruit new volunteers specifically for Trailblazer, and additional paid testers were recruited via the Prolific Academic platform (http://www.prolific.ac/, £7.50 per hour). We combined results from volunteers and paid participants as although paid testers are considerably faster than volunteers, the performance of the groups is not significantly different (data not shown). All participants provided informed consent to participation and storage of their data. The Health Research Authority approved this study (14/NW/1033). All participants that completed the test samples were included in the results reported here. None of the participants expressed any professional experience with pathology, but otherwise no demographic or data on educational achievement was collected. No participant participated more than once in any of the experiments.

### Tissue microarray samples

Samples from oesophageal and lung tissues were prepared as TMAs, immunohistochemically stained and imaged by the research groups of GJT and WH as previously described (Ward *et al*, 2014). The AK lab prepared the bladder cancer samples with p53 IHC as described previously (Cazier *et al*, 2014), and the bladder samples were stained with Ki67 using a clone MIB-1 (Dako, Agilent Technologies) at 1 in 1000 dilution on a Leica Bond machine, with Epitope Retrieval 1 buffer for 20 min. For all samples, patients consented to the use of their tissue for research (bladder cancer samples ethical approval 13/LO/0540; lung and oesophageal cancer samples ethical approval REC no. 10/H0504/32)

### Expert scoring

We obtained expert scores for the cancer detection task from 3 experts for all samples but lung/CD8, for which we had two additional experts. Three experts scored the IHC lung/EGFR sample and three experts scored the IHC bladder/p53 sample. The expert scores were provided through the same web interface used by the participants, except for the bladder samples which were scored as digital images in the lab. Pathologists entered their ratings independently from one another. Final expert consensus values, used to rate non-specialist participants were calculated as the majority vote (for cancer detection tasks) or the median value across experts (for IHC scoring of biomarker proportion and intensity).

### Online platform

All Trailblazer releases were developed using Pybossa, an open-source framework specifically developed for online crowdsourcing (https://github.com/PyBossa/). The stack consisted of Python, Django, Postgres, Javascript and jQuery. The platform was hosted on Amazon Web Services. Our code – available under a GNU Affero license – can be found at https://citizenscience.github.io.

### Detecting cancer cells

**Figure 1:**
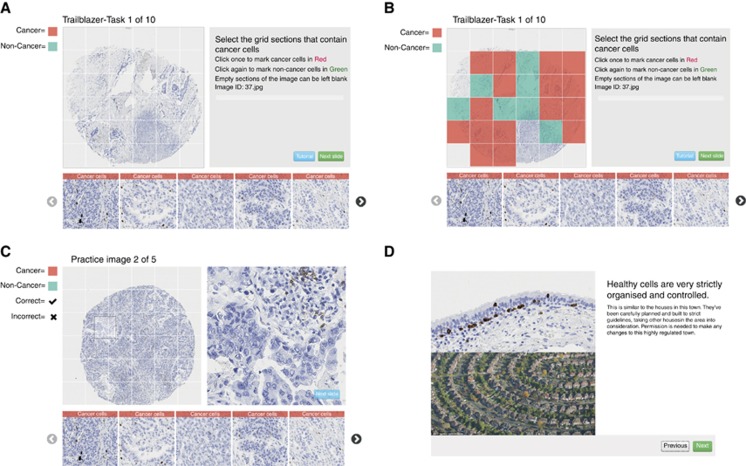
**The ‘Trailblazer' interface for viewing, annotating and scoring tissue microarray (TMA) cores.** (**A**) Participants evaluated squares on a 6x6 grid overlaid on a TMA for the presence of cancer cells. (**B**) They were asked to mark squares with cancer as red, cells without cancer as green and completely empty squares as blank. (**C**) To aid in cancer detection and IHC scoring, the participant could move their cursor over the core to reveal a high magnification view of the area under the cursor. Furthermore, a scrollable gallery of high magnification example images of cancer tissue and healthy tissue was available at the bottom of the screen. (**D**) Prior to starting the task each participant completed a ∼10-minute tutorial explaining the type of sample and how to distinguish cancer cells from non-cancer cells, of which a screenshot is shown here. In the first experiment we tested the effect of feedback-based training and/or annotated images provided in addition to this baseline tutorial.

**Figure 2:**
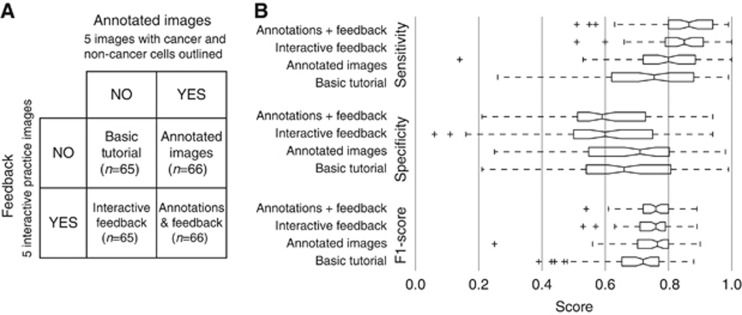
**Full factorial design to test the effect of annotated images and feedback-based training on cancer detection performance of individual participants.** (**A**) Experimental design and number of participants in each cell. (**B**) Box-plot graph showing performance in cancer detection across individuals in each of the four groups, expressed as F1-score, specificity and sensitivity. Statistics for main effects and interactions are shown in Table 1.

Participants were presented with a sequence of images and asked to identify regions where cancer was present. Ten images were overlaid by a 6 × 6 grid for a total of 360 squares (Figure 1). Participants then marked each square as containing no cancer (green), one or more cancer cells (red) or no tissue (blank; Figure 1B). A scrollable gallery of reference images illustrating a variety of cancer and non-cancer cells were included to aid correct analysis. The same ten images of lung cancer stained by CD8 (lung/CD8) were used throughout the testing of different tutorial mechanics. The ten images for each experiment were confirmed by consultant pathologists to be representative of the variety of possible tissue morphologies and biomarker staining patterns. The images were presented to each participant in a random order. We used a full factorial design (Figure 2A) to assess the effect of annotated images and feedback-based training in tutorials. The basic tutorial consisted of an ∼10- to 15-min, passive, text- and image-based set of instructions, developed based on interviews and training sessions with pathologists. Whilst all participants viewed the basic tutorial, they were randomly assigned to one of 4 groups in the factorial design. The tests investigated two additional tutorial elements. Firstly, the addition of 5 annotated images, shown to participants during the tutorial. Secondly, feedback-based training presented with 5 training images before the test images. For two images they were provided immediate feedback on each answer. For the remaining 3 images feedback was provided only after scoring the majority of the image. This was designed to mimic the learning experience of other successful crowdsourcing experiments (e.g., in EyeWire; Kim *et al*, 2014). The same five example images were used for both annotated images and feedback, and no images from the tutorial were used for testing. In addition to lung/CD8 a further three data sets were tested to confirm the accuracy of the tutorial including annotated images and feedback-based training.

### IHC biomarker scoring

Cancer detection is only the first step in TMA scoring; the next step is to score the percentage of cancer cells that are stained and the intensity of such staining. We therefore set out to test how accurately participants could score cancer staining, given the improved tutorial for cancer detection. We selected 21 lung/EGFR cytoplasmic stain samples and 30 bladder/p53 nuclear stain samples representative of the majority of clinical samples to test this, whereby each participant scored a random set of 10 images. These images were separate to the images used initially for cancer detection; no images from the tutorial were used as a test image. Participants indicated proportion of staining as a percentage, in increments of 5%. Where proportion was above 0%, i.e. stained cancer cells were present, the participant was asked to score staining intensity as 1 (weak), 2 (moderate), or 3 (strong). The product of these two, that is, a score between 0 and 300, is called the McCarty ‘H' score and is commonly used to relate IHC to patient outcomes and treatment response (McCarty *et al*, 1986). The tutorial for cancer detection was extended to explain IHC scoring, and users practiced IHC scoring through feedback-based training prior to scoring the TMA scores on which their performance was assessed. This extended tutorial, consisting of both a cancer detection and IHC scoring tutorial, took between 20 and 30 min to complete.

### Statistical analysis

Analyses presented in this paper are either at the level of individual participants or at the level of consensus ratings based on the aggregation of multiple participants. Whereas the former informs us about the effect of tutorial changes on individual performance, aggregated data underlies the power of crowdsourcing and is therefore the metric of interest when assessing the usefulness of this approach. During tutorial development for cancer detection, each participant provided 360 ratings (36 grid squares in 10 images). In the analysis we equated ‘blank' and ‘no cancer' responses such that each rating was binomial (positive or negative for cancer). We furthermore excluded 53 squares which contained no tissue whatsoever, as these would artificially boost the specificity. Each participant rating was then compared with the expert consensus on the basis of the presence or absence of cancer cells in each square. These comparisons were used to identify true positive (TP), true negative (TN), false positive (FP) and false negative (FN) responses from which sensitivity (TP/[TP+FN]), specificity (TN/[TN+FP]) and F1-score (2 × TP/[2 × TP+FP+FN]) were calculated (Figure 2). The general linear model was used to obtain coefficients and *P*-values on the main effects of feedback-based training and annotated images, and on their interaction. We computed Cohen's Kappa for each participant against the expert consensus, between pairs of experts, and for the participant consensus against the expert consensus.

One pertinent question in crowdsourcing is how many participants are required to provide accurate analyses for each image, with the underlying assumption of diminishing returns in group performance as more participants are added. We explored this question for both cancer detection and IHC scoring by bootstrapping various group sizes. We used the AUC described by the receiver operating characteristic – a common classification measure for a binary classifier – to assess group performance. Bootstrapping was used to estimate the accuracy of hypothetical groups between 3 and 40 participants in size. For a group size *n*, we sampled *n* participants from the complete population of participants with replacement, 500 times. Similarly, IHC scoring accuracy was assessed on the basis of Spearman *r* between the median expert score and bootstrapped groups of participants. For each image, we took the median of all responses for that image to calculate the aggregate H-score. IHC bootstrapping was performed using 10 000 samples.

All analyses were performed in Python using SciPy (Jones *et al*, 2001), scikit-learn (Pedregosa *et al*, 2011), scikits-bootstrap (https://github.com/cgevans/scikits-bootstrap), Pandas (McKinney, 2010) and NumPy (van der Walt *et al*, 2011). Graphs were created using Matplotlib (Hunter, 2007).

## Results

### Identification of cancer cells

In our first experiment we tested the efficacy of two tutorial elements such that participants could better distinguish cancer from non-cancer tissue. In the basic tutorial without annotated images or feedback, *individual* participants (as opposed to the aggregate of multiple responses which is more commonly used in crowdsourcing) achieved an average sensitivity of 0.74±0.04 (95% CI of the mean), specificity of 0.66±0.04, and F1-score of 0.70±0.03 (Figure 2B). We calculated main effects and interactions for the two factors using linear regression (see Table 1 for statistics). We found both annotated images and feedback-based training had statistically significant positive effects on the F1-score, with no interaction between the factors. In our experiment, adding both factors improved the F1-score by ∼0.05. Both tutorial components were therefore used in follow-up experiments. It is worth noting that the sensitivity-specificity trade-off was shifted strongly in favour of sensitivity in response to feedback-based training, whereas annotated images had no such effect (Table 1). In other words, feedback-based training lowers the threshold to indicate a square contains cancer.

### Cancer detection in different cancers and biomarkers

**Figure 3:**
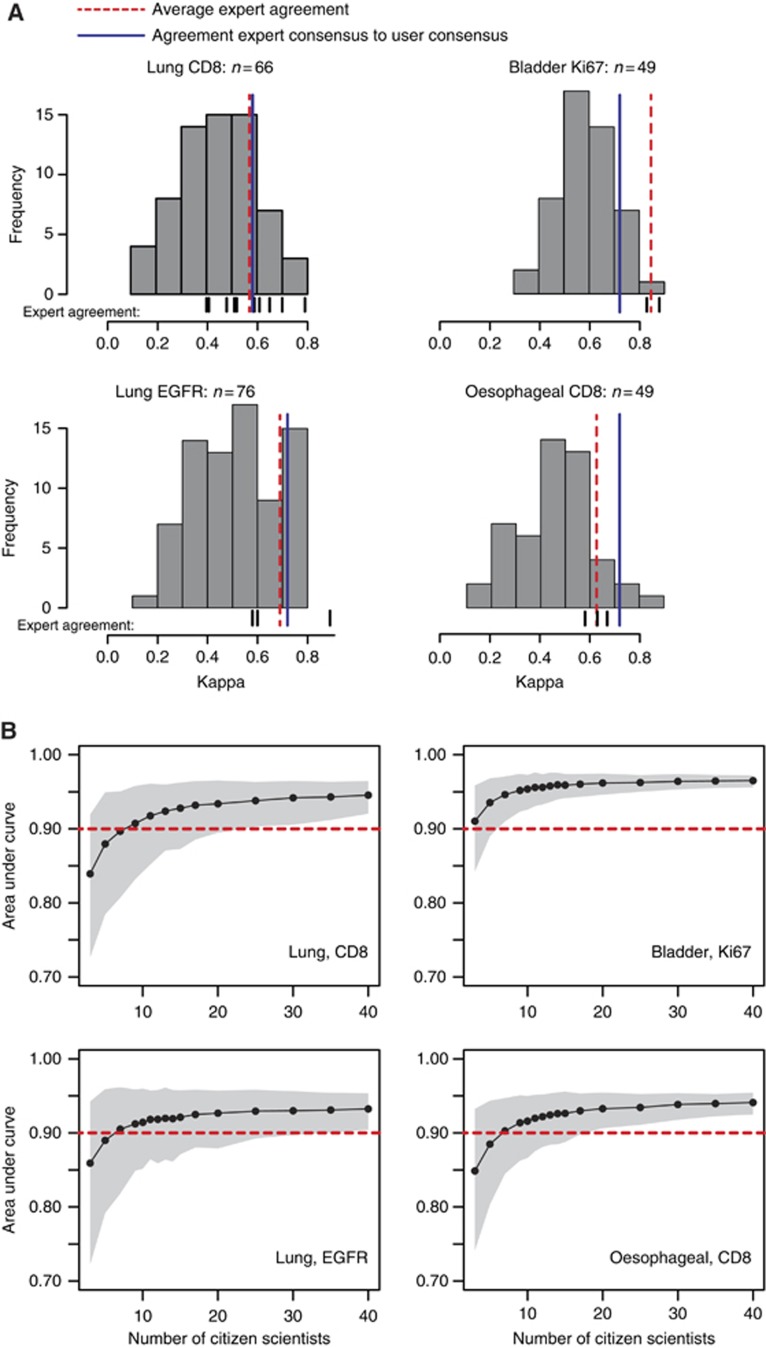
**Accuracy of aggregated responses across four sample types.** (**A**) We used Cohen's kappa to calculate correspondence between raters. The histogram indicates the distribution of kappas of each individual participant with the expert consensus. The solid blue line indicates the agreement between the majority consensus of all participants compared with the expert consensus, showing the majority outperforms the average individual. The pairwise kappas between experts are indicated as small black lines underneath the histogram; the average of the pairwise kappas is indicated in the dashed red line. (**B**) A second method to compare the participant consensus with expert consensus is the area under the receiver operating characteristic curve (AUC). Here we examined how the AUC changed as we varied the number of participants included in the consensus between 3 and 40. The red dotted line indicates an AUC of 0.90. Shaded areas indicate the bootstrapped 95th percentile CI.

We used the improved tutorial to test three additional data sets: a further set of lung samples stained for EGFR (*N*=76 participants), oesophageal samples stained for CD8 (*N*=49 participants), and bladder samples stained for Ki67 (*N*=49 participants). Critically, we now looked at both individual and aggregate performance, the latter by combining multiple cancer/no cancer responses for each individual square in the image. We first calculated Cohen's kappa for each participant with the expert consensus, revealing large differences between participants (Figure 3A). We then aggregated participants by calculating a majority consensus score for each square, which yielded ‘moderate' to ‘substantial' agreement (Landis and Koch, 1977) in each of the 4 sample types (Figure 3A). We also calculated the pairwise agreement between each of the experts and the average of those pairwise agreements. Strikingly, in 3 out of 4 sample types the majority consensus of participants was in better agreement with the expert consensus than experts among one another (Figure 3A).

A second way of quantifying performance of the aggregate group is to use the area under the receiver operating characteristic curve (AUC). Specifically, we were interested in the relationship between the number of participants evaluating a sample and the accuracy as measured by AUC. For each of the 4 sample types, we bootstrapped 500 samples for a number of participant population sizes between 3 and 40. In all cases the average AUC approached a maximum of ∼0.95 asymptotically as the number of participants per sample increased (Figure 3B). Altogether, in the majority of samples, a relatively small group of lay participants was able to approach levels of accuracy that would be expected from any one trained expert relative to another.

### Immunohistochemistry scoring

**Figure 4:**
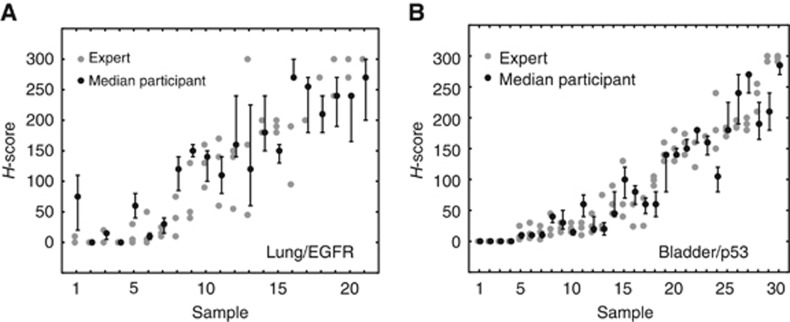
**Comparison of expert and aggregated participant H-scores for each image.** (**A**) Lung/EGFR sample. Grey dots indicate the three individual expert scores per sample, black dots indicate median H-score based on all participants who evaluated the image, error bars indicate the bootstrapped 95th percentile confidence interval of the median. The images have been sorted along the *x* axis by median expert score. (**B**) Bladder/p53 sample. For details described under (**A**).

**Figure 5:**
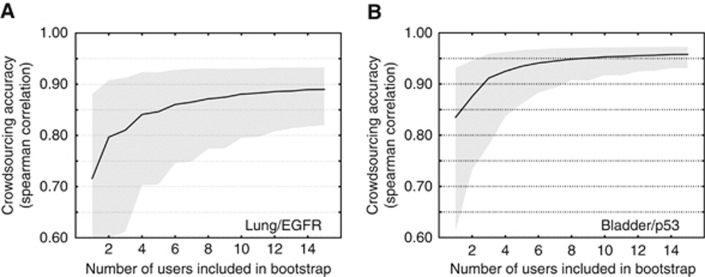
***H*-score accuracy as a function of number of participants evaluating each image.** (**A**) In lung/EGFR we observed that the Spearman correlation between participants and experts strongly increased as we included more participants in the aggregate score. The black line represents the median of the bootstrapped samples, and the shaded area represents the bootstrapped 95th percentile confidence interval of the median. (**B**) Bladder/p53, legend as in subplot a.

Having established tutorial elements that improve participant performance in the detection of cancer in TMAs and demonstrated that these permit high levels of agreement with experts in several different sample types, a key question remained: would the new interface yield reliable scoring of immunohistochemical staining in TMA samples? To answer this question, we tested IHC accuracy in the membrane/cytoplasmic marker EGFR in lung cancer (*N*=35 participants, each scoring 10 images) and for the nuclear marker p53 in bladder cancer (*N*=45 participants, each scoring 10 images). In the lung/EGFR data we observed a Spearman correlation of 0.91 (bootstrapped 95% CI=(0.78, 0.96)) between the median participant response and median expert score (Figure 4A). In the bladder/p53 sample, this same correlation was 0.97 (95% CI=(0.91, 0.99); Figure 4B). We also calculated how accuracy improved as we increased the number of participants evaluating each image (Figure 5). As was the case in cancer detection, having more than 5–10 participants rate each image did not yield substantial increases in group performance.

## Discussion

In this paper we addressed the hypothesis that crowdsourcing – distributing work to members of the general public – can be used to accurately analyse cancer TMA samples, using an online platform specifically developed for the clear presentation of samples. We initially examined the ability to distinguish cancer tissue from non-cancer tissue, a critical first step in IHC analysis, and found that annotated images and feedback-based training positively impacted on performance in lung/CD8 samples. We then applied this training method to three more sample types – lung/EGFR, oesophageal/CD8, and bladder/Ki67 – finding that aggregated responses from participants showed agreement with experts at a similar level as experts with one another, with AUCs between 0.90 and 0.95. Finally, we tested our improved tutorial for its usefulness in IHC scoring itself, finding strong correlations based on H-score between crowdsourced scores and experts. Altogether, these results provide evidence that the public can accurately analyse TMA samples, and suggest crowdsourcing as a potential additional resource to meet the growing demand for analysis resource in pathology research.

Our previous work in the analysis of breast cancer samples stained for oestrogen receptor showed an AUC of 0.95 for cancer detection at the whole core level, as well as strong correlations for IHC scoring with expert ratings (Cell Slider; Candido Dos Reis *et al*, 2015). However, this proof of principle was performed in the most common cancer and marker available, which can be analysed accurately using automated methods (e.g., Turbin *et al*, 2008; Bouzin *et al*, 2015; Howat *et al*, 2015). Here, we tested analytically challenging cancer types as well as immunohistochemical stains for which algorithms are either scarce or require considerable involvement from experts. By testing the crowdsourcing approach across a breadth of samples, we have shown this method to be flexible and widely applicable, including in sample types where algorithms struggle (Howat *et al*, 2015). Although both sample types we used for IHC scoring achieved high correlations with experts, the higher level of accuracy for bladder/p53 samples compared with lung/EGFR is most likely caused by the fact that the former is a nuclear marker whereas the latter is membranous. To our knowledge crowdsourcing has only seen limited investigation in cancer research. One study in renal cell carcinoma compared pathologists, research fellows, members of the public, and a fully automated algorithm on nucleus detection and segmentation (Irshad *et al*, 2014). They observed that members of the public performed similarly to research fellows, and either similarly to or better than the algorithm depending on the task.

Algorithms trained on large amounts of labelled data perform extremely well in many computer vision challenges (e.g., ImageNet; Russakovsky *et al*, 2015) including in cancer pathology (e.g., Walton *et al*, 2009; Beck *et al*, 2011). However, with over 200 cancer types and dozens of available immunohistochemical markers labelling different cellular components (nucleus, cytoplasm, and cell membrane) separately, obtaining sufficient training data for even a proportion of sample types is a considerable challenge. Crowdsourcing can provide a solution by scoring large data sets of samples for which no algorithms are available, and by subsequently making these data publicly available for researchers and commercial entities to develop automated methods. It is common practice for algorithms to supersede manual analysis in this way, as exemplified by the development of galaxy classifiers based on Galaxy Zoo data (Banerji *et al*, 2010), automated rather than crowdsourced analysis of electron microscopy data (Lee *et al*, 2015), and across the field of genomics. Our findings suggest such successes may be achieved on a large scale in pathology, where crowdsourcing can accelerate research by processing large volumes of samples currently being collected in clinical trials, as well as the vast amounts of tissue stored from past trials and routine archival material where patient consent is in place. Although crowdsourcing is not necessarily more resource-efficient than expert scoring – as it still requires ∼10 lay people to score each image to achieve accurate results – the sheer size of the general public and therefore the number of people that could potentially contribute to analysis provides a unique opportunity to accelerate research.

We set out to test two tutorial elements that might improve performance on the cancer detection task, and observed both annotated images and feedback-based training boosted overall accuracy. It has previously been observed that crowdsourcing can be improved by various means, including self-censoring of submissions when a user is uncertain of a response (Shah and Zhou, 2015), using videos rather than only text- or image-based instruction (Starr *et al*, 2014), having mini-breaks especially for complicated tasks (Rzeszotarski *et al*, 2013), presenting context-sensitive help (Andersen *et al*, 2012), and financial punishment for disagreement with other users (Shaw *et al*, 2011). Most research in crowdsourcing accuracy has been on paid workers, for example recruited through Amazon Turk. In the case of unpaid citizen science, however, users participate for non-financial reasons, primarily a desire to contribute to research (Raddick *et al*, 2013; Wright *et al*, 2015; Land-Zandstra *et al*, 2016) and to learn about science (e.g., Rotman *et al*, 2012). In such cases, offering financial incentives to improve accuracy would seem undesirable. Others have focused on improving the user experience to coax users to dedicate more time to the project, as experienced users are on average more productive than new users (Sauermann and Franzoni, 2015). All such tools, including our findings on tutorial optimisation, may be combined to establish crowdsourcing as an accurate tool for data analysis.

From this series of experiments, we conclude that crowdsourcing is an accurate and reliable analysis tool in TMA scoring – a major bottleneck in current clinical cancer research. We hope these results will encourage others in not only histopathology but cancer research more broadly, to take up crowdsourcing as a viable tool to analyse their data especially when the initial investment to set up crowdsourcing is outweighed by the ability to scale analysis (e.g., to segment 3D tissue samples; Booth *et al*, 2015). For those doing so, our open-source software can be used freely. Crowdsourcing in biomedicine is becoming more widespread (see for example https://citscibio.org/), and cancer research in particular stands to benefit a great deal from further investment given a combination of research need and strong public support.

## Figures and Tables

**Table 1:** Main effects of annotated images and feedback-based training and their interaction

**Factor**	**F1-score**	**Specificity**	**Sensitivity**
Annotated images	**β=2.11, (0.03, 4.20)** ***P*=0.047**	β=1.18 (−3.11, 5.48) *P*=0.59	β=2.69 (−0.64, 6.02) *P*=0.11
Feedback-based training	**β=3.14 (1.06, 5.23)** ***P*=0.003**	**β=−7.59 (−11.89, −3.30)** ***P*=0.001**	**β=8.77 (5.44, 12.10)** ***P*<0.001**
Interaction	β=−1.81 (−3.89, 0.27) *P*=0.09	β=−0.71 (−5.00, 3.58) *P*=0.75	β=−1.95 (−5.28, 1.38) *P*=0.25

All regression coefficients represent estimated change in performance when adding the factor, multiplied by 100. For example, adding annotated images is estimated to improve the F1-score by 0.0211. Values in brackets represent 95% confidence interval of the coefficient. Cells in bold are significant at *P*<0.05 uncorrected for multiple comparisons.
